# Epidemiology of congenital malformations in Tunisian liveborns: a retrospective study

**DOI:** 10.4314/ahs.v24i3.37

**Published:** 2024-09

**Authors:** Kaouther Nasri, Nadia Ben Jamaa, Mariem Aloui, Safouane Mansouri, Yosra Sdiri, Mariem Cheour, Samia Kacem

**Affiliations:** 1 Faculty of Sciences of Bizerte, University of Carthage, 7021 Zarzouna, Bizerte, Tunisia; 2 Department of Embryo-Fetopathology, Center for Maternity and Neonatology of Tunis, Tunis El Manar University, 1007 Tunis, Tunisia; 3 Department of Histology-Embryology, Faculty of Medicine of Tunis, Tunis El Manar University, 1007 Tunis, Tunisia; 4 Department of Neonatalogy, Center for Maternity and Neonatology of Tunis, Tunis El Manar University, 1007 Tunis, Tunisia

**Keywords:** Congenital malformations, liveborns, risk factors, prevention, Tunisia

## Abstract

**Objective:**

To identify the various congenital malformations in liveborns in the neonatology service within the center of maternity and neonatology of Tunis (CMNT).

**Methods:**

This is a retrospective study of liveborns with congenital malformations hospitalized during one year from 1rst January to 31 December 2016.

**Results:**

The profile of malformations was dominated by polymalformations (22.29%), followed by chromosomal aberrations (21.14%), cardiovascular malformations (16.00%), and system nervous malformations (11.43%).

Comparisons of liveborns and parental characteristics between all congenital malformations subtypes have shown significant differences in liveborns sex, consanguinity, and maternal age. Comparisons between malformed newborns and malformed fetuses have shown significant differences in consanguinity, rhesus type, maternal origin and parity.

**Conclusion:**

It seems important to set a careful surveillance of pregnancies at risk of developing congenital anomalies, systematic supplementation of vitamins and folic acid, and a national registry of congenital malformations.

## Introduction

Congenital malformations are very heterogeneous, of variable severity, ranging from simple disgrace without pathogenicity (minor malformation), to large malformations incompatible with life (major malformations). They are unique or multiple, primary (true) or secondary. Some are accidental and will not happen again. On the contrary, others have a genetic character which have to be specified to evaluate the risks of recidivism^1^.

The etiology of these malformations is multifactorial, determined by a set of genetic, infectious and environmental factors^2^, ^3^, ^4^.

Identifying these risk factors would help to reduce their incidence and therefore reduce the neonatal mortality rate, but it is often difficult to determine the exact cause. Congenital malformations are a real public health problem. According to the World Health Organization (WHO), three million newborns worldwide are born with major malformations, and about one-sixth of them die^5^.

Their prevalence varies significantly depending on the studied series and the concerned population. Some studies, which define the total and partial prevalence, involved only live children, others only stillbirths, and others only live children after termination of pregnancies. In the congenital malformation registry of Paris, the total prevalence is 3.2%, while the partial prevalence is 2.4%^6^.

Dorina C et al, conducted a study for three successive years (2011, 2012 and 2013) in the same hospital and concluded that the frequency and the nature of malformations are variable from one year to the next^7^.

In Chile, comparative studies between the years 1982 and 2010 have found a prevalence trend of 2.9% to 3.9%^8^.

At the international level, health professionals, governments and the general public have been made aware of the issue of congenital malformations, including the potentially teratogenic nature of drugs, following the tragedy of thalidomide in the 1960s^9^.

The establishment of surveillance systems for congenital malformations has become essential in many industrialized countries^10^.

Today, there are in France seven registers of congenital malformations qualified by the National Register Committee (NRC): the register of Paris, Alsace, Rhone-Alpes, Brittany, Reunion of Island, Auvergne, and the Antilles^11^.

Malformative census criteria, defined within the framework of the European Survival of Congenital Anomalies (EUROCAT) network, concern all malformations visible on clinical examination, visceral malformations, as well as all malformative syndromes identified or not, including those related to chromosomal abnormalities^12^.

The purpose of these registers is to monitor, on a continuous basis, geographically defined populations, in order to detect unexplained variations in the frequency of malformations and to alert the health authorities if necessary, but also to allow epidemiological studies to be carried out. Therefore, registry databases are an indispensable tool for public health research, especially when it comes to study the factors that may be involved in their occurrence, such as potentially teratogenic agents^10^.

In the absence of a national registry of congenital malformations, these tragedies may go unnoticed. In Tunisia, we deplore the absence of a national register to have a clear idea about the incidence of different anomalies, their distribution by region and their evolution over time. Only a few drafts of institutional registers have been attempted without much success.

In this work, we propose to identify the various congenital malformations in liveborns listed in the neonatology service within the center of maternity and neonatology of Tunis (CMNT) during the period of one year from 1^st^ January to 31 December 2016.

## Material and methods

This is a descriptive and retrospective study of liveborns with congenital malformations hospitalized in the neonatology department of the CMNT for a period of 1 year from 1^st^ January to 31^st^ December 2016.

## Patients

### Criteria for inclusion

All liveborns with one or more congenital malformations, who were admitted to the neonatology department of the CMNT, were included in our study.

Newborns who were alive at birth after termination of pregnancies were included.

### Criteria for non-inclusion

We did not include newborns who have a congenital malformation discovered at the outpatient clinic.

### Data collection

From the incoming staff notebook, we have listed all cases of congenital malformations suspected or confirmed; we collected 354 files. Records that contain an unconfirmed diagnosis, as well as records that were not found, have been removed. In total, we collected 175 files that met our inclusion criteria.

The information was collected from medical records using a medical file, to record the following data;

- Characteristics of the parents: origin, socio-economic level, consanguinity, antecedents of malformations, history of early death.
- Paternal characteristics: father's mean age.
- Maternal characteristics: mother's mean age, body mass index, blood group, medical history, gynecological and obstetric history, gestity, parity, dysgravidia, use of medicines during pregnancy.
- Liveborns characteristics: malformations profile, liveborns sex, frequency by birth month, evolution of study group liveborns, lifespan of deceased liveborns, malformations profile in deceased liveborns.

This study was also interested in a comparison of liveborns and parental characteristics between all congenital malformations subtypes discovered in this study. These differences were assessed using Chi-square tests by the statistical program SPSS v.18.

We also carried out a comparison of babies and parental characteristics between malformed liveborns and fetuses (aged between 8 and 36 weeks of gestations). Malformed fetuses data were obtained from our previous studies^2^, ^13^.

Differences in these distributions were assessed by binary logistic regression. To make statistical comparisons, 95% confidence intervals were reported. The rates were considered statistically significant at the 5% level (P < 0.05).

Ethical approval for the study was obtained from the Ethics Committee of the Maternity and Neonatology La Rabta Center in Tunis. All subjects gave written informed consent. Study participants consent for publication of their identifiable details, in relation to this article.

## Results

During this work, we studied 175 liveborns with one or more congenital malformations. They were hospitalized in the neonatology department of the maternity and neonatology center of Tunis during the year (1^st^ January to 31^st^ December 2016). In this study, we noted the presence of 12 liveborns after termination of pregnancy. A total of 15000 liveborns were born in the CMNT during the same year giving an overall prevalence of 11.66‰ in malformed liveborns.

### Characteristics of the parents

Characteristics of the parents were shown in Table 1.

**Table 1:** Characteristics of malformed liveborns parents (1^rst^ January-31 December 2016)

Characteristics	Percentage (%)
**Characteristics of the parents**	
***Origin***	
Greater Tunis	40.34
Outside Tunis	59.57
***Socio-economic level***	
Low	53.00
Intermediate	42.00
High	5.00
***Consanguinity***	
(+)	17.71
(-)	82.29
***Antecedents of malformations***	6.86
With malformed children	5.71
With a family history of malformations	1.14
***History of early death***	5.14
Single death	4.51
Two deaths	1.14
**Paternal characteristics**	
***Age***	
<35	32.21
≥35	67.79
**Maternal characteristics**	
***Young maternal age***	
≤19	1.26
>19	98.73
***Advanced maternal age***	
<35	66.45
≥35	33.54
***Body Mass Index***	
<25 kg/m2	50.00
25≤BMI<30	34.61
≥30	15.38
***Blood group***	
O+	45.33
A+	28.00
B+	15.33
B-	3.33
O-	2.67
AB+	2.67
A-	1.33
***Medical history***	
Type 1 diabetes	3.39
Chronic hepatitis B	1.12
Asthma	1.12
Hypothyroidism	1.12
Type 2 diabetes	0.56
Protein S deficiency	0.56
***Gynecological and obstetric history***	
Miscarriages	21.14
Abortion	4.00
Fetal death in utero	2.29
Intrauterine growth retardation	2.27
***Gestity***	
1	30.18
2	31.95
3	14.79
4	14.20
≥5	8.88
***Parity***	
1	67.00
2	50.00
3	34.00
4	15.00
≥5	11.00
***Dysgravidia***	26.29
***Use of medicines during pregnancy***	10.86

### Parental characteristics

The most frequent origin of the parents was from outside the capital of the country Tunis (59.57%). 40.34% of parents were from the capital Greater Tunis.

Parents of malformed liveborns were of predominantly low socioeconomic status in 53% of cases. Consanguinity marriage was present in 17.71% of cases.

I 6.86% of couples had antecedents of malformation, among them 5.71% had malformed children and 1.14% had a family history of malformations.

A5.14% of couples had a history of early death with 4.51% of them had a single history of infant death and 1.14% had two previous infant deaths.

### Paternal characteristics

The average father's age was 38.70 years with extremes ranging from 24 years to 56 years.

The presence of paternal pathological antecedents in our series was not noted.

### Maternal characteristics

The average maternal age was 32.20 years with extremes ranging from 17 to 46 years.

Body Mass Index (BMI) was mentioned in 14.85% of cases files. Body mass index less than 25 kg/m^2^ was predominant in 50% of cases.

The most common maternal blood type was O positive (45.33%), followed by A positive (28%).

Maternal medical history was noted in 22 cases with a predominance of Type 1 diabetes (3.39%).

21.14% of mothers had a history of miscarriages, 4.00% with previous abortions, 2.29% with fetal death in utero, and 2.27% with intrauterine growth restriction.

The average number of gestation was 2.22, with extremes going from G1 to G11. Mothers' cases were likely to have 2 gestations (31.95 %).

Parity ranged between 0 and 7, with an average of 2.09. Our results indicated that mothers cases were likely to be primiparous (67 %).

26.29% of pregnancies were complicated by dysgravidia. 10.86% of mothers had used one or more drugs during pregnancy.

### Liveborns characteristics

Liveborns characteristics were shown in Table 2.

**Table 2:** Malformed Liveborns characteristics

Characteristics	Percentage
**Liveborns sex**	
Male	53.68
Female	44.63
Asexual	1.69
**Frequency by birth month**	
January	6.21
February	9.60
March	9.60
April	6.21
May	10.73
June	7.90
July	8.47
August	7.90
September	9.03
October	9.03
November	6.77
December	8.47
**Evolution of study group liveborns**	
Deceased newborns	53.67
Living babies follow-up	22.59
to external consultation	
Living	15.25
babies transferred to other services	
Living	8.47
babies missed in external consultation	
**Lifespan of deceased liveborns**	
Early neonatal death	50.00
Late neonatal death	35.00
Death after 28 days	15.00

### Liveborns sex

Malformed newborns were more likely to be male (53.68%) giving a sex ratio equal to 1.23. The presence of 1.69% of asexual malformed liveborns was noted.

### Distribution of malformed newborns by birth month

The frequency and distribution of malformed new-borns by birth month during the year 2016 have shown a peak frequency in May with10.79% of malformed liveborns.

### Evolution of study group liveborns

The evolution of our study group liveborns was marked by the follow-up of 22.59% of living babies to external consultation, the transfer of 15.25% to other services, the loss of 8.47% in external consultation, and the death of 53.67% of cases.

### Lifespan of deceased liveborns

50% of cases have died in the early neonatal period (from birth to the 7th day of life), 35% have died in the late neonatal period (from the 8^th^ day to the 28^th^ day of life), and 10% died after 28 days.

### Profile of malformations

**Figure 1:**
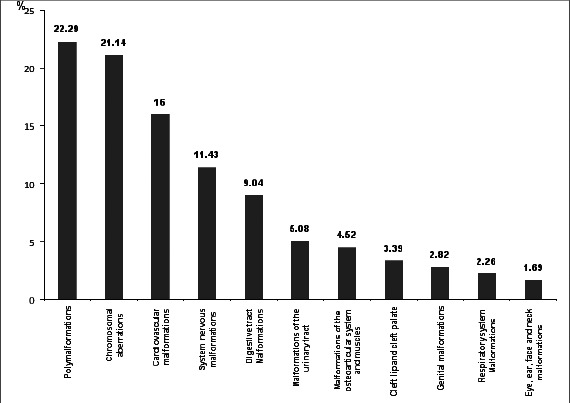
Malformations profile

As shown in Figure 1, the profile was dominated by polymalformations (22.29%), followed by chromosomal aberrations (21.14%), cardiovascular malformations (16.00%) and system nervous malformations (11.43%). Chromosomal aberrations were dominated by trisomy 21 in its homogeneous form in 24 cases. Cardiovascular malformations were dominated by complex cardiopathies. Nervous malformations was dominated by hydrocephalus.

**Figure 2:**
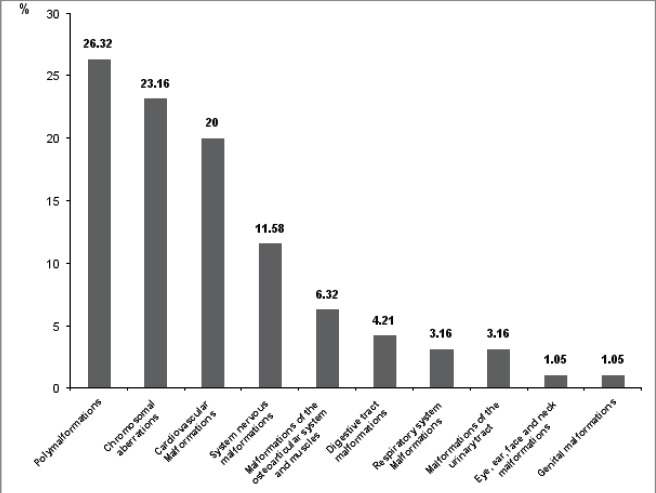
Malformations profile in deceased liveborns

Malformations profile in deceased liveborns was shown in Figure 2

MIt was characterized by 26.32% of deaths with polymalformations, 23.16% with chromosomal aberrations, 20% with cardiovascular malformations, and 11.58% with system nervous malformations.

### Distribution of liveborns and parental characteristics between all congenital malformations subtypes

As shown in Table 3, comparisons of liveborns and parental characteristics between all congenital malformations subtypes, have revealed a significant difference in liveborns sex (P= 0.047), consanguinity (P= 0.04) and maternal age (P= 0.042).

**Table 3:** Differences in distribution of liveborns/parental characteristics between all congenital malformations subtypes

Parental- liveborns characteristics (%)	1	2	3	4	5	6	7	8	9	10	11	P value
**Liveborns sex**												
Female	48.80	54.30	48.60	57.90	14.30	20.00	41.70	28.60	44.40	57.10	57.10	**0.047**
Male	51.20	45.70	48.60	42.10	85.70	80.00	58.30	57.10	55.60	42.90	42.90	
Asexual	0.00	0.00	2.70	0.00	0.00	0.00	0.00	14.30	0.00	0.00	0.00	
**Maternal pathology during pregnancy**												
(-)	77.80	69.70	77.40	88.90	100.00	90.00	87.00	50.00	88.20	71.40	85.70	0.19
(+)	22.20	30.30	22.60	11.10	0.00	10.00	13.00	50.00	11.80	28.60	14.30	
**Maternal pathology before pregnancy**												
(-)	84.20	97.10	87.90	78.90	85.70	100.00	69.60	91.70	88.20	85.70	76.90	0.25
(+)	15.80	2.90	12.10	21.10	14.30	0.00	30.40	8.30	11.80	14.30	23.10	
**Consanguinity**												
(-)	80.60	82.40	96.40	61.10	80.00	88.90	80.00	83.30	81.30	69.20	53.80	**0.04**
(+)	19.40	17.60	3.60	38.90	20.00	11.10	20.00	16.70	18.70	30.80	46.20	
**Multiple pregnancies**												
1	100.00	88.60	97.10	94.70	71.40	90.00	87.00	84.60	94.10	100.00	92.90	0.17
≥1	0.00	11.40	2.90	5.30	28.60	10.00	13.00	15.40	5.90	0.00	7.10	
**Parental socio-economic level**												
Low	46.20	55.60	93.80	66.70	50.00	25.00	53.80	44.40	55.60	50.00	57.10	0.23
Intermediate	46.20	33.30	6.20	22.20	50.00	25.00	46.20	44.40	33.30	40.00	42.90	
High	7.60	11.10	0.00	11.10	0.00	50.00	0.00	11.10	11.10	10.00	0.00	
**Gestity**												
1	18.40	47.10	27.80	21.10	28.60	20.00	21.70	38.50	35.30	35.70	21.40	0.38
≥1	81.60	52.90	72.20	78.90	71.40	80.00	78.30	61.50	64.70	64.30	78.60	
**Parity**												
1	34.20	58.80	36.10	31.60	28.60	30.00	34.80	46.20	41.20	42.90	28.60	0.58
≥1	65.80	41.20	63.90	68.40	71.40	70.00	65.20	53.80	58.80	57.10	71.40	
**Maternal age**												
<35	68.40	77.40	46.70	47.10	80.00	87.50	65.20	83.30	62.50	91.70	53.80	**0.042**
≥35	31.60	22.60	53.30	52.90	20.00	12.50	34.80	16.70	37.50	8.30	46.20	
**Maternal Origin**												
Greater Tunis	25.00	32.10	46.70	33.30	25.00	22.20	36.80	50.00	53.80	38.50	40.00	0.72
Other regions	75.00	67.90	53.30	66.70	75.00	77.80	63.20	50.00	46.20	61.50	60.00	
**Rhesus type**												
Rhesus -	11.40	3.40	9.10	0.00	0.00	0.00	9.10	15.40	0.00	0.00	0.00	0.55
Rhesus +	88.60	96.60	90.90	100.00	100.00	100.00	90.90	84.60	100.00	100.00	100.00	
**Antecedents of malformations**												
(-)	92.10	91.20	96.90	88.90	100.00	100.00	87.00	91.70	88.20	78.60	92.30	0.75
(+)	7.90	8.80	3.10	11.10	0.00	0.00	13.00	8.30	11.80	21.40	7.70	
**Paternal age**												
<35	28.00	16.70	15.80	30.00	0.00	20.00	23.10	20.00	11.10	16.70	28.60	0.98
≥35	72.00	83.30	84.20	70.00	100.00	80.00	76.90	80.00	88.90	83.30	71.40	

1: Polymalformations, 2: Chromosomal aberrations, 3: Cardiovascular malformations, 4: System nervous malformations, 5: Digestive tract Malformations, 6: Malformations of the urinary tract, 7: Malformations of the osteoarticular system and muscles, 8: Cleft lip and cleft palate, 9: Genital malformations, 10: Respiratory system malformations, 11: Eye, ear, face and neck malformations. (-) = Absence ; (+)= Presence ;

P value from chi-square test of the comparison between subtypes. The rates were considered statistically significant at the 5% level (P < 0.05) and marked in bold.

Malformed liveborns were significantly more likely to be male for the majority of subtypes (P = 0.047) especially in cases with digestive tract malformations (% = 85.70).

The rate of the absence of consanguinity was the most frequent for each subtype (P= 0.04) mainly in cases with cardiovascular malformations (96.40%).

Maternal age was compared in this part between two ranges (under and upper or equal to 35). The findings have revealed that mothers were aged less than 35 for each subtypes (P = 0.042) exceptionally the mothers of cases with respiratory system Malformations (91.70%).

### Distribution of liveborns and parental characteristics between malformed liveborns and fetuses

As shown in Table 4, comparisons of parental and babies characteristics between malformed fetuses and liveborns have demonstrated significant differences in consanguinity (P <0.0001), rhesus type (P= 0.04), maternal origin (P <0.0001) and parity (P <0.0001).

**Table 4:** Comparisons of parental and baby characteristics between malformed fetuses and liveborns (1^rst^ January-31 December 2016)

Parental-babies characteristics (%)	Malformed Liveborns	Malformed Fetuses	P value	OR	CI (95%)
**Fetal sex**					
Male	54.32	45.40	0.55	0.91	0.67-1.23
Female	45.76	47.50			
**Young maternal age**					
≤19	1.26	46.03	0.82	0.85	0.20-3.51
>19	98.73	47.60			
**Advanced maternal age**					
<35	66.45	47.06	0.12	0.76	0.54-1.07
≥35	33.54	47.78			
**Consanguinity**					
(-)	79.47	62.86	**<0.0001**	**0.45**	**0.30-0.67**
(+)	20.52	47.19			
**Maternal Rhesus type**					
Rhesus -	7.33	46.87	**0.04**	**0.47**	**0.30-0.88**
Rhesus +	92.66	48.55			
**Maternal origin**					
Greater Tunis	36.40	43.46	**<0.0001**	**0.38**	**0.25-0.58**
Other	63.57	51.11			
**Gestity**					
1	29.47	34.14	0.20	0.80	0.57-1.12
>1	70.52	65.85			
**Parity**					
≤1	38.72	58.42	**<0.0001**	**0.44**	**0.32-0.61**
>1	61.27	41.57			

(-)= Absence ; (+)= Presence.

o makes statistical comparisons, 95% confidence intervals were reported by binary logistic regression. The rates were considered statistically significant at the 5% level

(P < 0.05) and marked in bold.

Consanguinity was significantly more frequent in the parents of malformed fetuses (47.19%) than in the parents of malformed liveborns (20.52%).

Positive rhesus type was significantly more frequent in the mothers of malformed liveborns (92.66%) than in the mothers of malformed fetuses (48.55%).

The rate of malformed liveborns was significantly more important in the interior of the country (63.57%) than malformed fetuses (51.11%). Multiparous mothers were significantly more frequent in malformed liveborns (61.27%) than in malformed fetuses (41.57%).

## Discussion

In this current study, the prevalence of malformed liveborns during the period (1^st^ January-31^st^ December 2016) was 11.66 ‰.

The prevalence of malformed liveborns varied significantly depending on the studied series and population. This variation in frequency could also be explained by the variation of the methodological approach, criteria for inclusion or exclusion of minor anomalies, the size of the studied sample, the exact definition and classification of malformations and the access to complementary examinations to confirm suspected diagnoses.

Some studies have shown that the prevalence of malformations was increasing with time^3^, ^8^, ^14^. This increase might be due to the exploration of new techniques in live births and the practice of fetopathological examination in deceased malformed newborns.

The current facility of access to antenatal diagnosis has reduced the number of infant births with malformations. However, some of these pregnancies were insufficiently screened in utero, hence the interest of their detections and identifications. By this way, the detections of severe and complex malformations, or those associated with chromosomal abnormalities, allow to establish a therapeutic strategy in utero or at birth.

This is why, the study of certain epidemiological factors could determine a maternal population at increased risk of developing malformations.

In this study, the majority of malformed liveborns came from an unfavorable socio-economic background (53%). This finding was consistent with Bassil KL et al study in which the risk of malformed live births was increased one and a half times in couples whose socio-economic level was low in Canada^15^.

In our series, 17.71% of malformed newborns were born to a consanguineous marriage.

Several studies have considered inbred marriage as a risk factor for congenital malformations^16^, ^17^. According to the study of Nabulsi MM et al, this association might be due to that consanguinity increases the risk of polygenic recessive pathologies recurrence among progeny in cardiac malformations^18^.

The rate of malformed newborns from a consanguineous marriage were 7% in Mosayabi Z and Movahedian AH study^19^, 49.6% in Saudi Arabia according to the study of Majed-Saidan MA et al^20^, and 3% in Denmark according to the Corlnel study^17^.

This variability among countries could be explained by the cultural differences between these populations. In our study, the familial history of congenital malformation was present in 6.86% of cases. This frequency was lower than those found in Pakistan (22.12%)^21^, and Morocco (27.5%)^22^.

In our series, the average paternal age was 38.70 years old. This average age was higher than those in Canals CA and Sabiri N findings, which were respectively 34 and 35.3 years old^22^, ^23^.

Several studies have shown the absence of a relationship between paternal age and the prevalence of congenital malformations according to Zhu jl et al^24^ and Nazerhj et al^8^.

The mean maternal age was 32.20 years old with extremes ranging from 17 to 46 years. This result was consistent with the finding of a previous Tunisian study conducted by M'bazaa L^25^, but in contrast with Zemni study conducted at the CMNT in which lower maternal age was noted^26^.

As reported by the studies of Canals ca et al^23^ and Luoy l et al^27^, the rate of congenital malformations has increased with maternal age.

Advanced maternal age was implicated not only in chromosomal aberrations^28^, but also in several other malformations^29^, ^30^. This might be due to the aging of maternal gametes, as suggested by Hollier LM et al study^31^ in which congenital malformations unrelated to chromosomal aberrations were increased with a maternal age greater than 25 years, and those due to chromosomal aberrations were increased from the age of 35 years.

Maternal age under 20 years old was also involved in the nervous system, digestive system and osteoarticular system congenital malformations, according to Chen XK et al^10^.

In this study, 30.18% of the mothers were primigest. This finding suggested that a mother having her first child had a greater risk of developing fetal malformations, since the most frequent parity noted in this work was equal to 1 (Table 1). Our findings were consistent with the results of Luoy l et al^27^, and Zemni H^26^ studies.

Likewise, the study of Sarker S et al, have shown that 59.79% of mothers having a malformed newborn were primiparous^32^.

In contrast, McNeese ML et al have found that the risk of having a malformed newborn was rising with increasing parity and maternal age^33^.

When studying the medical history of mothers, the highest rate was noted for the mothers with Type 1 diabetes (3.39%). This result was consistent with the finding of Dong et al^34^. According to a previous Tunisian study, the overall incidence of diabetes in pregnant women was estimated to 5.1%^35^.

Maternal long-standing diabetes was also associated with the occurrence of congenital malformations. So, impaired maternal glycemic control for pre-existing diabetes increased this risk^36^, ^37^.

In this current study, 15.38% of mothers had a body mass index greater than 30 kilograms per square meter. Some studies have shown that maternal obesity was associated with the appearence of congenital malformations in fetuses^38^.

We have found that the sex ratio was 1.23 with a predominance of male (54.29%). 44.00% of malformed liveborns were female and 1.71% had an abnormality of sexual differentiation. This result was consistent with the findings of Richmoud S in Brittany^39^ and Mmbaga BT et al^40^ who reported a male predminance in their series.

This finding remains unexplained according to the same authors^40^.

57.71% of studied malformed liveborns were born at term. Singh K et al have found that 75% of malformed liveborns were born at term^41^, while some studies have noted that malformed newborns were predominantly born prematurely^21^, ^32^.

IWe have noted that 54.29% of malformed newborns have died. This rate was higher than those found in the Abdi-rad J et al and Sabiri N et al series which reported respectively 38.26% and 5% of deaths^22^, ^42^.

This high mortality rate might be explained by the raised prevalence of polymalformations, cardiovascular malformations, neurological malformations and lethal chromosomal aberrations such as Trisomy 13 and 18 in our group (Table 2).

Several newborns have died due to the lack of spaces in intensive care.

To better understand the association between parental/liveborns characteristics and the occurrence of the different malformations in liveborns, the present study evaluated differences in the distribution of specific malformations subtypes by parental/liveborns characteristics in Tunisia during (1^st^ January-31^st^ December 2016) (Table 3).

This comparison has shown a significant difference in liveborns sex (P= 0.047), consanguinity (P= 0.04) and maternal age (P= 0.042).

Our findings have revealed that mothers were aged less than 35 years for each subtypes (P = 0.042) exceptionally the mothers of cases with respiratory system malformations (91.70%). This result was in contrast with others studies which found that maternal age above 30 years had a significant impact on the emergence of fetal malformations like spina bifida and down syndrome^14^,^28^. According to the WHO, advanced maternal age increased the risk of chromosomal abnormalities, including Down syndrome, while younger maternal age increased the risk of certain birth defects^43^.

According to Bugnon and his collaborators, the risk of the birth of a malformed infant was increased from the age of 30 and has become major beyond the age of 35. This has been mainly due to the aging of gametes, especially female ones. This hypothesis has been based on the fact that oogenesis begins with a very long meiotic prophase and that the oocytes produced at the end of genital life contain a higher proportion of chromosomal abnormalities^44^.

In this current study, comparisons of parental/babies characteristics between malformed fetuses and liveborns have shown a significant differences in consanguinity (P <0.0001), maternal Rhesus type (P=0.04), maternal origin (P <0.0001) and parity (P <0.0001).

These significant differences might be due to the severity of malformations in fetuses. Fetal malformations were lethal and led to fetal death in utero or a justified indication for a medical termination of pregnancy. While the malformations detected in liveborns in the present study were less serious (53.67% deaths).

Consanguinity was significantly more frequent in the parents of malformed fetuses than in the parents of malformed liveborns. This indicates the role of this risk factor in very severe and lethal malformations which need a medical termination of pregnancy during the first weeks.

The rate of malformed liveborns was significantly more important in the interior of the country than malformed fetuses. This might be due to the difficulty of access to antenatal diagnosis in these regions. This is why, malformed infants were transferred to our center after birth in order to manage their malformation.

Multiparous mothers were significantly more frequent in malformed liveborns than in malformed fetuses. This finding might be the result of unmonitored pregnancies which could be more frequent in multiparous mothers of malformed liveborns. This has prouved the important rate of undiagnosed prenatal malformations for mothers of malformed liveborns from the interior of the country suggested previously.

## Conclusion

Congenital malformations are a major cause of morbidity and mortality. The identification of risk factors specific to each country allows specific prevention measures. The surveillance of pregnant women, education of young women of childbearing age and monitoring of pregnancies should be public health priorities.

Despite improved management of malformed liveborns, they remain burdened with significant morbidity and mortality. This is why, it seems important to set a rigorous and careful surveillance of pregnancies at risk of developing congenital anomalies, systematic supplementation of vitamins and folic acid for all pregnancies, and a national registry of congenital malformations.

## Abbreviation

CMNT: Center of Maternity and Neonatology of Tunis
EUROCAT: European Survival of Congenital Anomalies
NRC: National Register Committee
WHO: World Health Organization
